# Web-browser encryption of personal health information

**DOI:** 10.1186/1472-6947-11-70

**Published:** 2011-11-10

**Authors:** Richard E Morse, Prakash Nadkarni, David A Schoenfeld, Dianne M Finkelstein

**Affiliations:** 1 MGH Biostatistics Center, Massachusetts General Hospital, 50 Staniford St. Ste 560, Boston, MA, 02114, USA; 2 Yale University, New Haven, CT, 06511, USA

## Abstract

**Background:**

Electronic health records provide access to an unprecedented amount of clinical data for research that can accelerate the development of effective medical practices. However it is important to protect patient confidentiality, as many medical conditions are stigmatized and disclosure could result in personal and/or financial loss.

**Results:**

We describe a system for remote data entry that allows the data that would identify the patient to be encrypted in the web browser of the person entering the data. These data cannot be decrypted on the server by the staff at the data center but can be decrypted by the person entering the data or their delegate. We developed this system to solve a problem that arose in the context of clinical research, but it is applicable in a range of situations where sensitive information is stored and updated in a database and it is necessary to ensure that it cannot be viewed by any except those intentionally given access.

**Conclusion:**

By developing this system, we are able to centralize the collection of some patient data while minimizing the risk that protected health information be made available to study personnel who are not authorized to use it.

## Background

Confidentiality of patient information has become a major issue in the storage and retrieval of health care data and biological samples. Some diseases, such as AIDS and depression, are stigmatized, and patients are often concerned that they could lose their employment or their insurance coverage if these conditions are revealed. Similarly, stored biological samples can be used to identify future health risks, impacting a patient's chance of obtaining insurance. As the move is made to electronic storage of all health information, it is crucial that a patient's identity be protected in health databases. At the same time, a medical record is dynamic and it is important that a health care team be able to unambiguously identify a patient when they need to retrieve or update the information in the record.

The Privacy Regulations [1] released under the Health Insurance Portability and Accountability Act of 1996 (HIPAA) [2] define different levels of confidentiality of a data set. The most confidential is a data set which includes *protected health information *(PHI) that can unambiguously identify a patient. Such a data set should only be available to a patient's care-givers and the institutions that work with them to provide care. A lower level of confidentiality is provided by a *limited data set*, which does not contain names, addresses, or social security numbers, but may contain information such as birth date, geographical region, and dates of hospital admission -- data that could allow a patient to be identified in a hospital population. A limited data set can be transferred to a research group with a written legal agreement between the care-giving institution and the research group. In general, care-giving institutions only allow the transferal or collection of limited data sets with the patient's consent. A *de-identified *data set is the least confidential type of data set, and is supposed to have all data which can identify the patient removed from it. In practice, HIPAA defines particular classes of data which must be removed (e.g. names, dates, and geographical information more specific than zip code). However, using modern data mining techniques, patients may still be identifiable from a de-identified data set. De-identified data sets may be used by research groups without restriction and can be posted on the internet.

**Figure 1:**
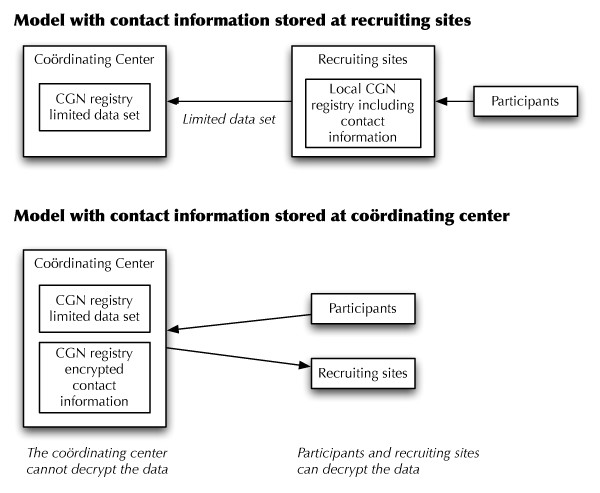
**CGN data organization**. This figure shows how the data storage for the CGN registry was re-organized in 2007.

The issue of encrypting *protected health information *arose because the MGH Biostatistics Center is the coöordinating center for the National Cancer Institute's Cancer Genetics Network (CGN). The CGN is a multi-institution research network organized with the goal of creating a registry of people with an elevated cancer risk who could be approached to participate in research studies on genetic susceptibility and the related psychological and health outcomes. When the network was founded in 1999, a de-centralized model was used wherein each participating institution (or *site*) recruited participants from the site's local clinics and registries. Each participant completed a survey of their medical history, and were recontacted annually by the enrolling site for updates in this data and their contact information. The data was collected at each site into a local database, and a limited data set was abstracted from the local database and uploaded to a central merged database held by the coöordinating center [see figure 1]. The contact information was never sent to the central site; the consent form signed by the participants explicitly stated that the contact information would only be available to staff at their enrolling site.

In 2007 the CGN decided that there was the potential to improve the efficiency and cost of the network's activities by moving to a centralized model, where the sites were no longer responsible for storing the medical and contact data. By doing this, we would also be able to develop a system to allow the option for participants to update their data online, thus reducing the workload on the sites. Participants who chose not to update their data online would continue to be contacted by the sites for follow-up. Those who used the online system would have their updates go directly into the central database.

Centralizing the medical and family history data was straight-forward, as this was merely a change in where the data was entered: instead of sites entering data into their local database and sending the coördinating center the abstract, they would enter data directly into the central database. However, centralizing the contact information was problematic due to the consent form that the participants had signed. In order to transfer the contact information to the coördinating center, the sites' institutional review boards (IRBs) would require re-consenting all participants to allow the coördinating center staff to have access to the PHI. This would be costly and would likely cause considerable attrition of participants. Discussion with the sites determined that the consent allowed us to store the contact information provided that no coöordinating center staff could access the information. The ability to use the data to contact the participants would have to remain with the individual sites.

Any system we developed would have to make it *impossible *for staff at the coördinating center to access, understand, or make use of data that could be used to identify the individual patient. This included IT staff, database administrators, and web developers. At the same time, this identifying information would need to be easy to access and update by both the participants and project staff at the sites. Thus, we could not require installation of any software on the participants' computers. It would also have to be possible for the sites to retrieve and decrypt the information in bulk.

## Implementation

### Overview

**Figure 2:**
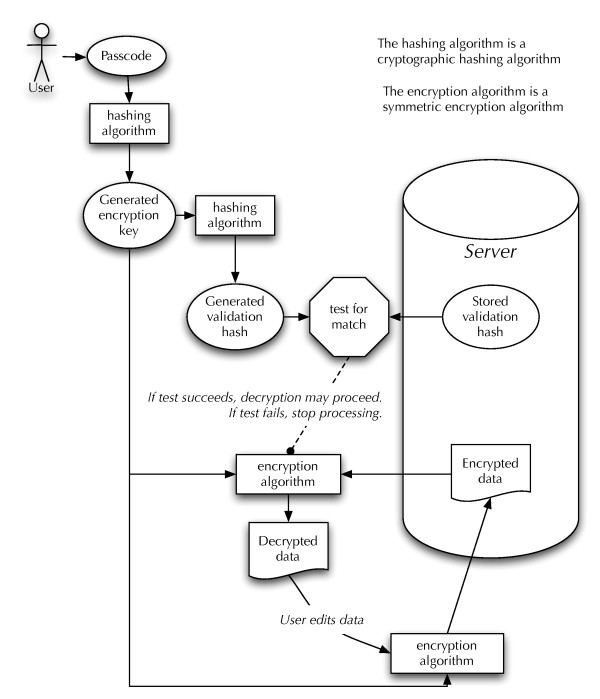
**Encryption data flow**. A diagram laying out how the encrypted data and the user-supplied passcode are used to decrypt the data.

The purpose of this system is to allow encrypted data to be decrypted in a web-browser client and to re-encrypt it before being submitted to the server. This allows *protected health data *to be stored without allowing support personnel on the server to use the data. At no point is the encryption key stored on, or made available to, the server. Instead, the server stores a cryptographic one-way hash of the encryption key, which the client uses to ensure that the provided encryption key will properly decrypt the data. Figure 2 provides an overview of the data flow for the system.

### Constraints

The CGN uses TrialDB, a web-based clinical study management, data capture, and storage system [3] to capture data on the participants. TrialDB is a highly customizable system which offers many advanced features including a data-element library, dynamic case report forms, complex data validation within and between forms, as well as skip logic and built-in support for controlled vocabularies such as DSM-IV, ICD-10, etc. In order for our system to be useful, we had to integrate it into TrialDB. TrialDB allows the injection of completely external forms, so that forms which are not already supported can be created and used. Through this facility, we were able to implement this system within TrialDB without needing to modify TrialDB itself. We hope that a future version of TrialDB will include this encryption system or a similar one.

The contact information needed to be accessible both to the project staff at the sites and to the participants, as we wanted to allow the participants to update their own data. This constraint ruled out the use of a public/private key solution since the infrastructure required to set up such a system for 26,000 participants and 15 sites would be unmanageable by the coördinating center. Thus we decided to use a symmetric encryption algorithm: the Advanced Encryption Standard (or AES; sometimes also called Rijndael), in large part because it is a government standard [4].

AES requires an encryption key of either 128, 192, or 256 bits. Requiring participants (and, to a lesser extent, staff at the sites) to enter a 256-bit encryption key in order to access their contact data is only slightly less infeasible than managing a large public/private key network. To solve this problem, we decided to use SHA-256, a cryptographic hashing algorithm, to expand a "passcode" to a 256 bit encryption key.

Early tests indicated that people would often mistype the passcode, and the system would decrypt the data into gibberish. Participants especially, but also staff at the sites, would then correct the information and save it, encrypting it using the wrong passcode. This would then render the data useless.

We could not make the passcode or the encryption key available to the server for verification as that would defeat the purpose of the system. However, cryptographic hashing functions are usually "trapdoor" functions: you cannot retrieve the input to the function given the output; in particular, SHA-256 *is *such a function. We can use SHA-256 to generate a "verification hash" from the encryption key. This value *can *be stored on the server since it is not possible to get back to the encryption key from the verification hash. We can then transmit this to the client along with the encrypted data, which can verify that the passcode, when hashed twice, is transformed into the verification hash. If it is, then the passcode (and, thereby, the encryption key) is correct, and decryption can proceed.

Although it isn't directly related to the system, one final concern was that a site might somehow forget or lose their passcode. Our solution to this was the use of an escrow service. The service could be given a copy of the passcode, encryption key, and verification hash in a sealed envelope. However, it would not have access to the database (which requires a separate username and password from the encryption passcode).

An initial step to the use of this system is that all of the protected health information needs to be encrypted and sent to the central database. We developed separate programs to provide this functionality. For more information on these programs, as well as how we allow sites to retrieve their data in bulk format and decrypt it easily, please contact the authors.

What follows is an implementation of the webbrowser based encryption system we have been discussing. The system used in production is slightly different; the largest difference being that instead of using the "prompt" command to request information from the user, we use a JavaScript-based dialog box. This makes the code more complex as it breaks up much of the logic into various callbacks instead of having a linear flow. The few other changes between the code discussed below and the one we use are related to changes in JavaScript libraries between 2005 and now.

Our original implementation was done in 2005 as a demonstration. The actual application was started in late 2007.

### Components

We use the following third-party components in this system:

• A Javascript AES implementation. There are several implementations available online. We use the one from movable-type.co.uk [5].

• A Javascript SHA-256 implementation (technically this is one of the SHA-2 family of hashing functions). We use jsSHA2 [6], slightly modified to put it into a namespace. We have also incorporated some of the changes from webtoolkit.info [7]. The Webtoolkit implementation is derived from jsSHA2 and contains several useful changes (especially for UTF-8 strings), but it was easier to make jsSHA2 work with Windows Scripting Host, which was required for other reasons not relevant to this paper.

• jQuery [8] is a Javascript library that makes working with the web browser DOM much easier. Although not strictly necessary, it makes the code much cleaner, and easier to get working across browsers. Our original implementation predates jQuery, but the production version uses jQuery.

Also, although we don't use it in this paper, we use jQuery Impromptu [9] for a Javascript-based dialog box in our actual system.

### Code

A complete listing of the HTML sent to the webbrowser is in table 1. This same HTML, including referenced libraries, is available in additional file 1. For this system to work in practice, a server-side program is also needed which connects to a data store and provides the encrypted data and the verification hash to the client.

**Table 1:** Code listing

1	<html>
2	<!-- This code is provided under the terms of the Creative Commons
3	"Attribution (CC BY)"license. Please see
4	http://creativecommons.org/licenses/for details -->
5	<head>
6	<title>Client-side Encryption </title>
7	<script type="text/javascript"src="jquery-1.4.2.min.js"></script>
8	<script type="text/javascript"src="sha256.js"></script>
9	<script type="text/javascript"src="aes.js"></script>
10	<script type="text/javascript">
11	//verification hash for passcode "passcode".
12	//this would be provided from the database.
13	var GLOBAL_verification_hash
14	= "a983169676c9fdcc852f29c013aef5f6faac345dfff472548bf93a543618d5ed";
15	var GLOBAL_passcode =null;
16	var GLOBAL_encryption_key =null;
17	
18	/**
19	*assume that all encrypted fields start encrypted
20	*/
21	$(document).ready(function () {
22	$(".encrypted").each(function () {
23	$(this).attr("encryption_status", "encrypted");
24	});
25	});
26	
27	/**
28	*get the passcode
29	*/
30	$(document).ready(function () {
31	if ($(".encrypted").length >0) {
32	try {
33	if (!GLOBAL_verification_hash) {
34	throw "There is no verification hash available.";
35	}
36	
37	GLOBAL_passcode =prompt("Please enter your encryption passcode:", "");
38	if (!GLOBAL_passcode) {
39	throw "You did not enter an encryption passcode.";
40	}
41	
42	GLOBAL_encryption_key =SHA256.hex_sha256(GLOBAL_passcode);
43	
44	if (SHA256.hex_sha256(GLOBAL_encryption_key) !=GLOBAL_verification_hash) {
45	throw "The passcode you entered does not verify. "
46	}
47	
48	decrypt_data();
49	}
50	catch (error_message) {
51	$(".encrypted").attr("disabled","disabled");
52	alert(error_message + "Encrypted fields will be disabled.");
53	}
54	}
55	});
56	
57	/**
58	*on form submission, encrypt the data
59	*/
60	$(document).ready(function () {
61	$("#encryption_form").submit(function () {
62	encrypt_data();
63	return true;
64	});
65	});
66	
67	/**
68	*find all encrypted encryptable fields and decrypt them.
69	*use Base64 to keep characters in the printable range.
70	*/
71	function decrypt_data () {
72	$(".encrypted[encryption_status=encrypted]").each(function () {
73	var encrypted_text = $(this).val();
74	if (encrypted_text != "") {
75	var plain_text =AesCtr.decrypt(Base64.decode(encrypted_text),
76	GLOBAL_encryption_key, 256);
77	$(this).val(plain_text);
78	}
79	$(this).attr("encryption_status", "decrypted");
80	});
81	}
82	
83	/**
84	*find all decrypted encryptable fields and encrypt them.
85	*use Base64 to keep characters in the printable range.
86	*/
87	function encrypt_data () {
88	$(".encrypted[encryption_status=decrypted]").each(function () {
89	var plain_text = $.trim($(this).val());
90	if (plain_text != "") {
91	var encrypted_text =Base64.encode(AesCtr.encrypt(plain_text,
92	GLOBAL_encryption_key, 256));
93	$(this).val(encrypted_text);
94	}
95	$(this).attr("encryption_status", "encrypted");
96	});
97	}
98	
99	</script>
100	</head>
101	<body>
102	<form id="encryption_form">
103	Encrypted: <input type="text"
104	name="enc_field"
105	id="enc_field"
106	value="TkVjM1RENCtQajR6ZUIzZktRY0VrdndQVTB6Q091akQvTHpjeVE9PQ=="
107	class="encrypted"/><br/>
108	Unencrypted: <input type="text"
109	name="field"
110	id="field"
111	value="some unencrypted value"
112	class=""/><br/>
113	<input type="submit"value="Encrypt and submit"/>
114	</form>
115	</body>
116	</html>

This is the complete HTML code listing referred to in the "Implementation" section above.

The HTML source is comprised of three main sections. Lines 7-9 load the external libraries mentioned above. Lines 10-99 are the encryption and decryption code. Lines 102-114 are an example form, with both an encrypted field and a un-encrypted field. The data for the input fields of the form needs to be supplied by the server.

There are three global variables that we use. These are defined in lines 11-16. The GLOBAL_verification_hash variable would be provided by the server; in this case we have hard-coded the verification hash for a passcode of 'passcode'.

This system supports mixing encrypted and unencrypted data. Data that should be decrypted for editing and then encrypted upon submission must be in an input field with the class "encrypted". Any field which doesn't have this class is ignored by the system, and passed to the server as-is on submission.

When the page is loaded in the browser, we use the $(document).ready() idiom provided by jQuery to run three functions, defined in lines 18- 65. This idiom ensures that the page is completely loaded before we execute the function. They are executed in the order that they are added to the jQuery object using the ready() function.

The first function (lines 18-25) attaches a dynamic property to each encrypted field to indicate the encryption status. This prevents double-encrypting or double-decrypting. We assume that all encrypted fields came from the server encrypted.

The second function (lines 27-55) prompts the user for the encryption passcode and decrypts any encrypted fields. Line 31 ensures that the user is only prompted for a passcode if there are encrypted fields in the form. There are several possible error conditions that can occur. In order to simplify the logic, we use a "try/catch" block. If an error occurs, the encrypted fields are disabled, so that the data in them cannot be corrupted. The key steps in this function occur in lines 42-48. On line 42, the encryption key is generated from the user-supplied passcode. On line 44, the encryption key is hashed a second time, and compared to the GLOBAL_verification_hash. If they match, line 48 calls the function to decrypt the data.

The final $(document).ready() function (lines 57-65) attaches a validation function to the submit handler of the form which ensures that the data is encrypted before it is sent to the server.

The encryption and decryption of the data is fairly straight-forward. We use two helper functions, decrypt_data (lines 67-81) and encrypt_data (lines 83-97) to keep the $(document).ready() functions neater. Each of these functions iterate over all of the encryptable fields that are not currently in the incorrect state. Because the result of the encryption process is not necessarily safe -- it might contain quote characters, or angle brackets, or odd characters outside of the normal printable characters -- the encrypted text is encoded using Base64, which is included with the AES library we are using. Before we can decrypt the data, of course, we need to decode the field value. Note that each function changes the encryption_status attribute for the fields. This ensures that they don't run twice on the same field in the event that they get called twice in a row.

## Discussion

When we were developing this system, encryption in the browser was novel -- there were very few implementations available, and many of them existed only in order to show that it could be done. At the time, we looked for any examples of similar systems online, and could not find any. Since then, we have learned of a few systems which are similar in some ways (e.g. the Drupal module "Client Side Encryption" [10]) but such projects are unusual. Encryption of sensitive data is common, but encryption and decryption usually happen on the server (see, for example, [11]) and therefore do not work for our use-case.

The use of a verification hash is a technique we have not seen elsewhere. This is the key feature of the system that allows us to permit the general public to use the system without a risk of massive data corruption.

This description and our implementation of this system is targeted towards protected health information. In our case, we have consent to have access to a limited data set, so it is not necessary to de-identify the data. However, this system could be adapted to transform limited or protected data sets into de-identified data sets. Besides contact information, the most common sensitive data points are dates, such as dates of admission or treatment (which are not allowed in a de-identified data set). Using this method of client-side decryption, it would be possible to have a base date stored in an encrypted manner. All other dates could be stored as offsets from this base date. When the form is displayed to a care team, the browser-client can add the offsets to the base date, and display actual dates. When the data is used for analysis, only the offsets are available. The base date need not even be a relevant date; any base would suffice.

It is important to note that this system is only intended to protect data from misuse on the server side of the system. This is intended to be used as a part of a larger system which provides user authentication, access logs, and other protections against unauthorized access from clients.

One criticism that has been suggested with our particular implementation is that we use a single passcode for all of the participants at each site. This allows the staff at the site to access all of the data, but does somewhat limit the ability to say that the passcode is "secret". In our usage, this is not important (all that matters is that the coöordinating center staff never learn the passcode) as access to the data is also protected by a separate username and password pair -- the encryption passcode is only used to protect the contact information. However, there is a relatively simple enhancement that obviates this concern. When the data is initially encrypted, instead of using one passcode, two passcodes could be supplied. One is the "site" passcode, which is shared for all participants at a site. The other is the "participant" passcode, which is unique to each participant. The participant passcode would be used to encrypt the data. The participant passcode (or its generated encryption key) is then encrypted using the site passcode. The database stores the participant verification hash, the encrypted participant passcode, and the site verification hash. Participants can then use their passcode to access the data. For site staff, the site passcode is used to decrypt the participant passcode, which then is used to decrypt the data.

This system was devised for the encryption of protected health information. It is possible to determine if an encrypted value is completely missing, as the field in the database will be empty, but other data validation checks, such as range checks or cross-question integrity, cannot be performed on the server. Many of these kinds of checks can be performed using client-side JavaScript before the encryption takes place, but most of the kinds of fields that this system would be used for are likely to be fields that cannot be checked without human intervention; this kind of validation would need to be performed by a person who has permission to use the decrypted data.

## Conclusion

In this paper, we have presented a web-browser based system that uses client-side encryption to prevent sensitive data from being available to personnel that should not have access. Although this solution seems straight-forward, we spent several weeks looking for existing technologies that offered all of the features that we needed, and did not find any.

The most interesting part of the system is the use of a verification hash to ensure that the encryption key is correct without the server being able to decrypt the data. This was a very important step that allowed us to use the system with the general public without concern about data corruption.

Although we developed this system for a specific situation, there are interesting directions it can be taken. Any situation where specific data needs to be available to a care team or to data-entry staff, but should not be available to central staff or to an IT team, could benefit from this technique. As privacy issues become more challenging, such a system could simplify many of the data-sharing issues between institutions doing multi-center clinical trials.

## Availability and requirements

The main code for this system is available under the Creative Commons Attribution license. The AES library is also available under the Creative Commons Attribution license. jsSHA2 is available under the BSD license. The Webtoolkit modifications are licensed under the Creative Commons Attribution 2.0 UK license. jQuery is available under the MIT license or the GPL.

In order to use this code, you need a web-browser. This has been tested in Safari, Firefox, and IE6+.

## Competing interests

The authors declare that they have no competing interests.

## Authors' contributions

REM was the primary architect of this system. PN supplied critical feedback during development, and his past experience with TrialDB and data entry led to several important changes and refinements. DS was involved in the initial impetus to develop the system and gave important feedback for the writing of this paper, including suggestions that led to a change of focus and several of the ideas for other ways to use the system. DF requested this application, and provided guidance and feedback on the manuscript preparation. All authors have read and approved the final version of this manuscript.

## Pre-publication history

The pre-publication history for this paper can be accessed here:

http://www.biomedcentral.com/1472-6947/11/70/prepub

## Supplementary Material

Additional file 1 **The web site**. The sample website, including the JavaScript libraries, as a. zip file. Click here for file
