# Supplementary material for: Web-browser encryption of personal health information
Source: BMC Med Inform Decis Mak. 2011 Nov 10;11:70. doi: 10.1186/1472-6947-11-70 (PMC3276430; doi:10.1186/1472-6947-11-70)
Supplement: Additional file 1 — The web site. The sample website, including the JavaScript libraries, as a. zip file. [file 1472-6947-11-70-S1.ZIP › encryption_site/index.html]

Client-side Encryption


Encrypted:   
Unencrypted:   
